# Traditional Medicine in the Pristine Village of Prokoško Lake on Vranica Mountain, Bosnia and Herzegovina

**DOI:** 10.3797/scipharm.1003-06

**Published:** 2010-04-26

**Authors:** Broza Šarić-Kundalić, Elisabeth Fritz, Christoph Dobeš, Johannes Saukel

**Affiliations:** Department of Pharmacognosy, University of Vienna, Althanstraße 14, 1090 Vienna, Austria

**Keywords:** Traditional medicine, Ethnobotany, Bosnia and Herzegovina, Vranica, Prokoško Lake

## Abstract

The results of an ethnobotanical study conducted in the pristine village of Prokoško Lake (Vranica Mountain, Bosnia and Herzegovina) in summer 2007 is presented. Informal interviews involving 12 informants known as “traditional healers” provided data from 43 plants used in 82 prescriptions. The applied plants were used for a broad spectrum of indications. The most frequent were gastro-intestinal tract ailments, blood system disorders, skin ailments, respiratory tract ailments and urinary-genital tract ailments. The most frequent preparation was an infusion. Other often used preparations were ointments or balms and decocts. The special Bosnian balms known as “mehlems” were prepared from freshly chopped or freshly pressed herbal parts of various plant species. Warmed resins from *Abies* or *Picea* species, raw cow or pig lard, olive oil and honey served as basis. The traditional doctors, who usually worked as a team, enjoyed such a good reputation that people from all over the country were visiting in search of alternative ways to cure their ailments and diseases. The practical techniques applied by the healers and some of their attitudes and values are reported.

## Introduction

Traditional medicine is as old as human kind and is practised by virtually all cultures, each one with its own indigenous knowledge, health practices and beliefs. Scientific and “non-scientific” knowledge generally has been transmitted by oral tradition from generation to generation since antiquity until it finally became a significant part of the foundation of today’s school medicine. The history of traditional use of drugs for healing, hence, dates back far to ancient civilisations, for instance to those of Babylon, Egypt, antique Greece and to the Romans. Slavs arrived in Europe in 7^th^ century. However, it had been the immigrating Ottomans at the end of 15^th^ century who built the first hospitals, called “Meristans”, on the Balkans [1]. Due to insecure and bad roads in those times, sick people rarely visited the usually remote hospitals. As people practically only had limited access to hospitals, these institutions could not seriously develop as an alternative to traditional medical practices. Consequently, knowledge about the medicinal benefits of plants persisted in the population. The so called “herbal doctors” entered the stage. The first hospitals meeting today’s standards on the Balkans opened much later at the beginning of 19^th^ century [1]. Nevertheless, use of traditional plant medicine didn’t decline. In the 20^th^ century it was claimed that people kept with traditional medicine for financial reasons. However, still after the 2^nd^ World War and even today, when medical care became free in hospitals, many people kept with folk medicine. Especially during the last decades, due to another war shaking the Balkans, their countries including Bosnia and Herzegovina saw a revival of traditional medicine. The limited availability of proper medical care forced people during these years to revert to traditional medical practices to meet their health care needs. In particular herbs served as alternatives. During these severe times, the book of the Bosnian famous herbs collector Sadiković [2] was the only found reference on traditional use of plants from Bosnia and Herzegovina available to the people. It was not until 2007, that Redžić published the first “systematic” study about the ethnobotanical use of wild plants in Bosnia and Herzegovina [3]. Additional work elaborating on this topic together with a literature discussion is currently under way and will be published elsewhere [4, 5].

Compared to countries of Central and Western Europe, traditional medicine still is largely underexplored in Bosnia and Herzegovina, making this botanically exceptionally diverse country [6] a prime target for ethnobotanical and ethnopharmacological research. Although the history of this country indicates that traditional medicine in Bosnia and Herzegovina has Ottoman roots, which explains the observed similarities in medical practice, the obvious floristic differences among these countries promoted the development of unique applications specific to Bosnia & Herzegovina.

In order to get insights into traditional practice and knowledge, we will explore a pristine region in Bosnia, the area of Prokoško Lake. We are going to systematically collect information about the usage of wild plants from the inhabitants of the Village Prokoško Lake. For this purpose, we will gather information on verbally delivered prescriptions and indications, the historical background of applications as well as of the acting people, and the procedures applied to prepare the plants. Special attention will be paid to endemic species and preparations typical for this region. The relevant information will be mainly obtained from interviews of people known as „traditional healers“ by the inhabitants of the village. Based on these data, finally, the practical organization, structure and function of the medical system will be described and a survey of wild plants used in traditional medicine of this village provided.

## Materials and methods

### Geographic, historical and political context of the study

The republic of Bosnia and Herzegovina forms a triangle with an extension of 51,129 km^2^ located in the western Balkan Peninsula. Bordered to the north, west and south by Croatia (border length 932 km), to the east by Serbia (302 km), and to the south by Montenegro (225 km), Bosnia and Herzegovina is almost landlocked, except for the short Adriatic coastline of about 26 km near the town of Neum in the outermost south-west.

The country of Bosnia and Herzegovina is dominated by mountains and hills covering approximately 66% of the total area. Nature is highly diverse as demonstrated by the occurrence of 3,572 different vascular plant species, 3,000 species of algae, and 3,000–5,000 fungi and lichen species. Considering the relative small size of the country, these numbers make Bosnia and Herzegovina one of the five richest countries in Europe in terms of species diversity [6].

The eventful history of Bosnia and Herzegovina exposed the country to numerous cultural influences by different nations, starting with the Illyrians and Romans, followed by the Slavs and finally the Ottomans who occupied this territory in 1463 and made it part of their Empire for the following four centuries [7]. In 1878 the Congress of Berlin put the Ottoman provinces Bosnia and Herzegovina under the rule of Austria-Hungary It were the Austrian public officers who eventually coined the compound name Bosnia-Herzegovina, which remained the name of the country till today [8]. Following World War I Bosnia and Herzegovina was part of the South Slav Kingdom of Serbs, Croats and Slovenes (soon renamed Kingdom of Yugoslavia). The history and development of Bosnia and Herzegovina as member of socialistic Yugoslavia after World War II and until 1992 was then closely bound to the politics of this federal republic under the leadership of Josip Broz Tito [8]. In the early 90ies a war with Serbia shaked Bosnia and Herzegovina killing more than 200,000 people. In 1995, according to Dayton’s peace agreement, two separate governing entities were established, the Federation of Bosnia and Herzegovina and the Republika Srpska, but still joined under one roof, the country of Bosnia and Herzegovina [8].

According to a census, in 1991 Bosnia and Herzegovina had a population of 4,377,033 composed of the following ethnical groups: 1,902,956 (43%) Bosniaks, 1,366,104 (31%) Serbs, 760,852 (17%) Croats, and 242,682 (6%) Yugoslavs. The remaining 3% of the population – numbering approximately 104,439 people – consisted of various other ethnicities [9]. According to unofficial data of 2,000, Bosnia's largest ethnic groups are the Bosniaks (48%), Serbs (37%) and Croats (14%) [10].

### Area under investigation

The famous mountain Vranica belongs to the Dinaric Alps and stretches between the city of Fojnica in the east and Gornji Vakuf in the west. The massif of Vranica Mountain extends with many hills, pastures, surface waters and couple of peaks above 2000 m and is more than 100 km^2^ in size. Alongside with Zec Mountain it is part of a Paleozoic core. The highest peak of Vranica, the Nakrstac (2,012 m), represents the third highest peak of the country. In order to conserve the flora and fauna of Vranica Mountain, in 2008 this region was declared „a protected area due to its unique characteristics specific to the Federation of Bosnia and Herzegovina“.

The study was carried out in the Village of Prokoško Lake. The lake Prokoško Lake, which shares its name with the village, is of glacial origin and located on Vranica Mountain at about 1,660 m. It was declared a protected zone (“monument of nature”) by law in 2005 because of its exceptional plant diversity including a variety of endemic species. The lake is surrounded by traditional *Katunis* (Bosnian Shepard’s Huts) which form a small village where people live like in ancient times. There doesn’t exist electricity, mains water and modern medical care facilities. The health of inhabitants, as well as of tourists, depends on the traditional healers.

### Collection of data

Data were gathered by performing so called open ethnobotanical interviews [11] in summer of 2007.

In applying this method the following data were systematically collected:

- name, age and occupation of the interviewed person
- geographic locality and date of interview
- common name of the used plant
- part of the plant being used
- prescription background
- preparation procedure
- indication

### Identification of the collected plant material and data processing

Most of the plants used by the healers were observed in situ and collected for taxonomic identification in company with the informants. The collected plant material (more than 200 herbarium vouchers) was determined by the authors using floristic treatments covering Bosnia-Herzegovina [12–29]. Some of the species were ratified by means of book images or authentic descriptions of plants by the informants themselves. Voucher specimens were deposited at the Herbarium of the Department of Pharmacognosy, University of Vienna (WUP).

For further analyses and comparisons (e.g. comparison of usage of wild and cultivated plants used in traditional medicine of Austria and Bosnia and Herzegovina) assembled data were entered in the so called “VOLKSMED” data base of Austrian prescriptions [30, 31].

## Results and discussion

In 2007 in scope of a bigger field ethnobotanical and ethnopharmacological investigation of Bosnia and Herzegovina, traditional medical practice was studied in detail in the Village of Prokoško Lake. According to the informants and the literature record [3] the area was investigated in this respect for the first time. The research program was carried out through three consecutive days. For the first two days the authors gained permission to study the ways by which „traditional healers“ consulted their patients and how they prepared drugs. During breaks interviews were performed. In total, 12 out of the about 90 inhabitants could be questioned. The interviewed people were members of different ethnical groups, although the majority (8 of these 12 people) were Bosniaks (Bosnian Muslims). Informants were of an average age of 72 years. The third day was spent collecting plants used for the applied preparations. Understandably, the traditional healers didn’t provide their knowledge freely. It wasn’t before assuring them, that the provided information won’t be passed on to other “traditional healers” and that their valuable information will be used for scientific purposes only, that they agreed to share their medicinal methods and preparations with us. However, it has to be assumed that the healers did offer part of their knowledge only.

### Practices and values of traditional medicine in the Village of Prokoško Lake

The healers usually worked as a team, they conferred with each other and worked jointly. They also specialised on particular aspects and activities. Some were responsible for consulting the patients (usually six older ladies), others for preparing needed medicines (4 younger ladies) and some were experts in collecting plants (2 older man). The healers unified the abilities of a doctor, pharmacist and botanist.

Instead of written prescriptions, patients either got preparations ready to use like macerates, tinctures, sirups, balms from the doctors or – if requested – received a single plant or mixture along with an orally delivered instruction providing details on the preparation procedure and the proper application of drugs. Most of the preparations had to be made freshly as they otherwise would spoil soon – especially in summer – outside of a refrigerator which is not available without electricity. Only time-consuming and stable preparations like macerates or tinctures were prepared in larger amounts and stored in dark rooms. Many patients, therefore, had to wait for their medicines to be finished and often stayed over night in cottages reserved for tourists and guests. One night in such a cottage costs approximately moderate 2,50 € per person. The healers themselves had separate cottages, but consulting and preparation of medicines took place in one particular house with two separate rooms and slightly bigger than the other cottages. In the bigger room the healers consulted the patients and – in a compartment separated by a canvas – made the preparations. The second, smaller room served as storage for preparations and other products like self-made honey as well as for the purpose to dry plants.

Quite interesting appeared the way of payment, as healers didn’t charge fixed prices for their efforts, but left this decision with the patients. The system built on mutual trust and honesty. It relied on the judgment and satisfaction of the patients and on the abilities and readiness of the healers. This attitude went well with the practiced custom, that healers didn’t wish to get the money put into their hands, but instead asked the patients to secretly hide it under a cloth lying on a table nearby.

The needed utensils were self-made from wood or sheet. Mortars and pestles used to grind and mix substances were made out of wood, different cups and dishes for cooking, on the contrary, from sheet. Hoppers consisted of both wood as well as of sheet, whereas rakes used to collect berries, as an exception, were also cut from plastic (usually from used plastic bottles). Finished preparations were always stored in glass vessels including cups, bottles and other forms. Depending on the bottled substance, clear or dark glass was preferred. Glass utensils were cleaned in hot water and then sterilized by heating in an oven before use.

Medical care was ensured in particular for people who didn’t have access to treatments by academic or “modern” doctors. The “traditional doctors” thereby enjoyed such a good reputation, that people from all over the country were visiting in search of alternative ways to cure their ailments and diseases. Patients came from quite different parts of Bosnia and Herzegovina, some of them (3 out of 18 in 2 days) arrived even from neighboring Croatia and Serbia. The ethnicity or occupation of the patients accordingly differed and it seemed that it didn’t matter that some of the preparations included Muslim prayers. Visits were motivated by various goals. Tourists came by while relaxing and enjoying the nature of the area. Some just wanted to satisfy their curiosity and usually bought a “feel good” tea, a relaxing bath mixture or a mild sedative. Patients who came purposeful for medical reasons had very different health problems. Among them were even people plagued by serious diseases and symptoms like a bad blood picture after a chemotherapy or young women with sterility problems.

Even today, superstition played a dominant role in the beliefs of the healers. They separated their belief in God (healers were in general religious) from superstition, which has been passed over from generation to generation since ages just the same way as they handed down their medical knowledge. Superstitious and non-superstitious aspects, however, interwove with each other in the medical treatments. A good example for the importance of superstitious beliefs in healing were nervous system disorders which were considered “bad luck” or “devils work”. Interestingly, the treatment of such diseases was exclusively up to two old ladies. “Saljevanje strave” was the name of the healing method practiced, actually a ritual carried out by these women. Melted lead was poured into a bowl of water placed above the patient’s head. The causes underlying the nervous system disorders were than inferred from the appearance of the solidified lead. This procedure was at the same time supposed to free the patient from the identified bad influences. Amulets made of small pieces of paper with prayers written on them were also used to deal with these kinds of mental problems.

Other stories persisting in the village and defying logic or scientific knowledge at least were on medicinal herbs. The beliefs in the origin of formic acid in stinging nettles of the genus *Urtica* can serve as an example: The locals reported, that stinging nettles obtained their healing power from visiting ants as a reward for protection by these well-fortified plants (formic acid means also ant’s acid). We also recorded different sayings concerning plants supposed to have supernatural properties as vividly expressed in the following example: “Trava Iva od mrtva pravi živa”, (“Mountain germander [=*Teucrium montanum*] makes a dead man alive”).

### Plant species, preparations and medicinal use

In this study 82 prescriptions applied in human therapy could be collected in scope of the 12 interviews performed. All prescriptions constituted traditional knowledge as in the past they have been delivered verbally from one generation to the other, usually from mother to daughter, some reaching back as far as to 1817 [11]. Forty-three species used by the informants are listed in Table 1 together with their scientific names, affiliation to the botanical family, local common names, English names, the plant parts used as well as the medical indications and preparation procedures. Of particular interest appeared species which are unknown or rarely used by traditional medicine in Central Europe (cf. Austrian traditional medicine; [30]) as for instance species endemic to the Balkans. More attention should be paid to these plants as some of them may be of therapeutic value. Out of these we would like to mention in particular *Achillea nobilis, Artemisia laciniata, Teucrium montanum, Picea glauca, Urtica galeopsifolia,* and the endemic species *Teucrium arduini, Satureja montana*, and *Satureja subspicata* [4].

The applied plants were used for a broad spectrum of indications (see Table 1 for details). The most frequent ones were gastro-intestinal tract ailments (mentioned 41 times by the informants), blood system disorders (mentioned 36 times), skin (mentioned 35 times), respiratory tract (mentioned 23 times) and urinary-genital tract ailments (mentioned 20 times). Less commonly treated were rheumatism (mentioned 20 times), disorders of the metabolism and the nervous system (both mentioned 10 times) as well as disorders of senses, cardio-vascular system, gall and musculoskeletal system, each of which were reported less then 10 times by the informants.

The most frequent preparation was an infusion (tea, about 35% of the recorded preparations), mostly used internally. In a couple of cases only, the tea was used externally as a flushing fluid. Infusions were followed in frequency of appliance by ointments or balms as well as by decocts each used in about 18% of the cases. All other preparations were found to be less important as they were mentioned less then 11 times by the informants (approx. 5.5%). These preparations were directly applied fresh plants or freshly pressed juices, tinctures, powders, sirups, macerates, oils and collars. Water, alcoholic fluids (red wine, white wine and hard liquor, so called Bosnian “šljivovica”), vinegar and milk served as solvents. Honey played an outstanding role in traditional medicine of this people. It was applied as preservative agent or as an additive of cosmetic products as well as a sweetener in teas [4].

The special Bosnian balms known as “mehlems” were prepared from freshly chopped or freshly pressed herbal parts of various plant species. Warmed resins from *Abies* or *Picea* species, raw cow or pig lard, olive oil and honey served as basis. The plants most frequently used for these balms were species of the genera *Arctium*, *Carlina*, *Euphrasia*, *Hypericum*, *Plantago*, *Teucrium* and *Urtica* [11]. The balms were applied to cure skin and other external injuries or against rheumatism, and in the case of *Euphrasia rostkoviana* to treat eye injuries.

## Conclusions

“Those who cannot remember the past are condemned to repeat it” [32]. The body of existing ethnomedical knowledge has considerably contributed to great developments in healthcare. With the rapid industrialization of the planet and the associated loss of ethnic cultures and customs, traditional knowledge disappears. An abundance of ethnomedical information on plant uses exists in the scientific literature but often has not yet been compiled and made available for practical use. Systematic collection and compilation of ethnomedical information remain primarily academic endeavors. The presented study provides such a survey of traditional medical plant use on the example of the pristine Bosnian village Prokoško Lake. We recorded a considerable vitality of traditional plant use in this area, expressed by a wide range of indications and prescription reported by the local healers. A high number of medically used plants and of novel common plant names was encountered. Nevertheless, since informants were selected from among people known for their wide knowledge of plant uses and were in average 72 years old, we conclude that folk phytotherapy is “aging”. Knowledge of medicinal plants persisted mainly in elderly rural people who usually do not teach. The transmission of traditional medical knowledge from generation to generation is now threatened in this region which bears the danger of its disappearance. This study was a first but non-exhaustive contribution to the ethnobotany of the Village of Prokoško Lake. Due to its high botanical diversity and the large body of still existing traditional knowledge, this region has an exceptional pharmacological potential and, hence, for further ethnobotanical and ethnomedical studies. More research is necessary to reach at a more complete picture of the existing ethnobotanical knowledge, including all kinds of useful plants, not only in Prokoško Lake but also in other pristine regions of Bosnia.

## Figures and Tables

**Fig. 1.**
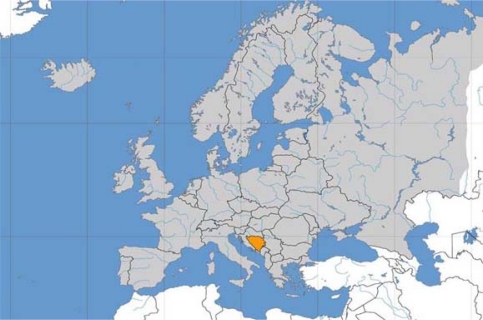
Map of Bosnia and Herzegovina

**Fig. 2.**
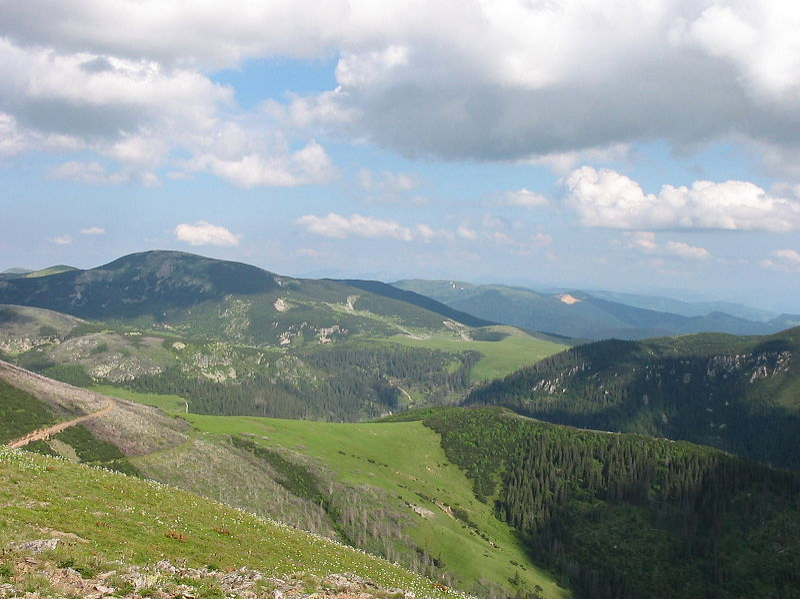
Vranica Mountain (from www.wikipedia.org)

**Fig. 3.**
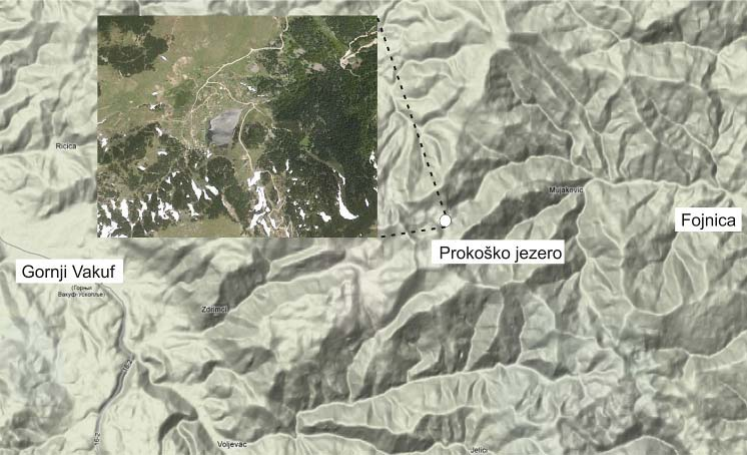
Area under investigation: satellite view of Prokoško Lake

**Fig. 4.**
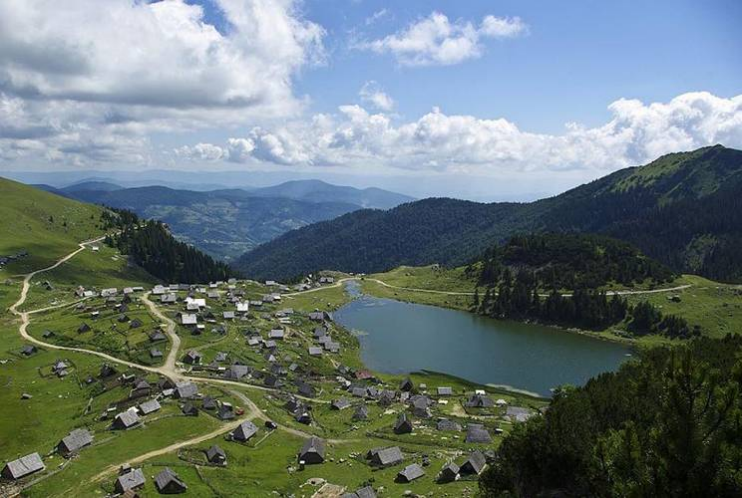
Area under investigation: birds view of Prokoško Lake, village and lake (from www.wikipedia.org)

**Fig. 5–8:**
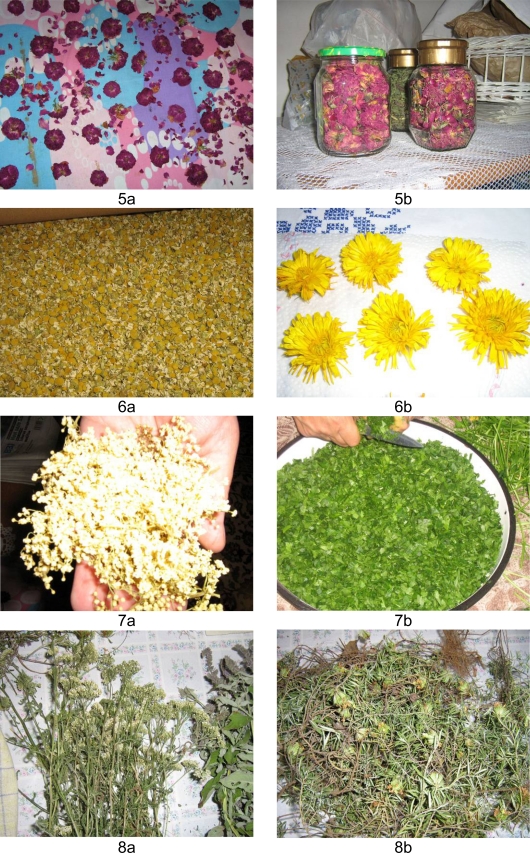
Drying of plants

**Fig. 9.**
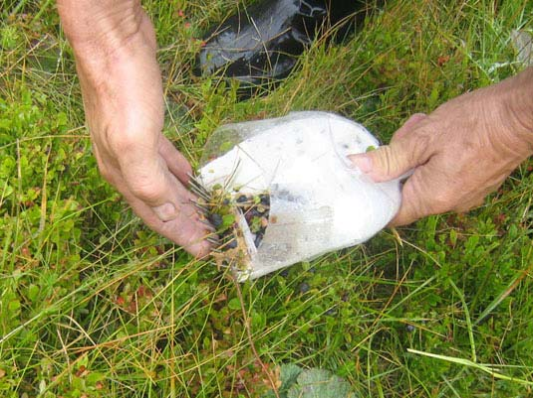
Plastic rakes used to collect berries

**Fig. 10.**
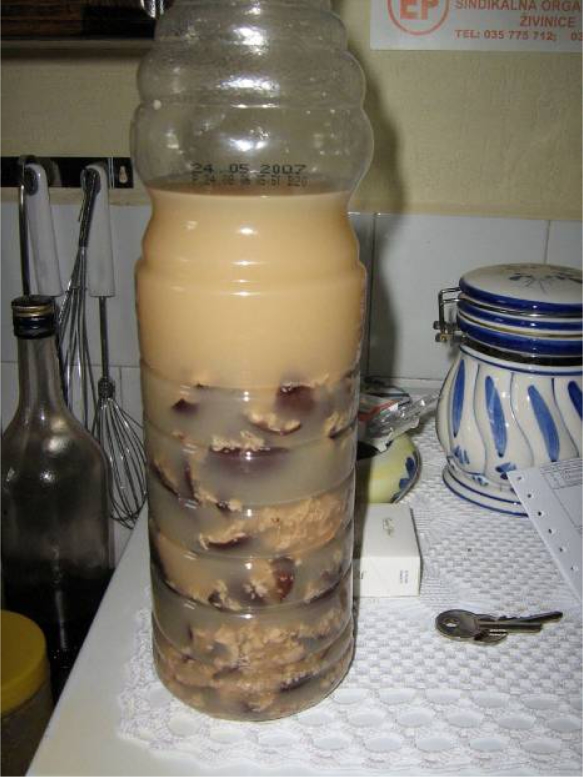
Horse chestnut tincture

**Fig. 11.**
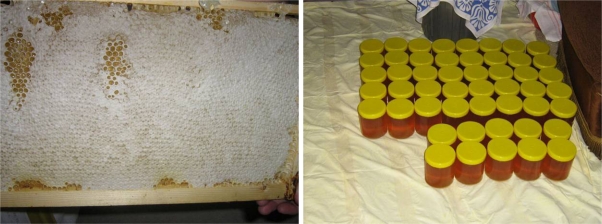
Self made honey

**Fig. 12.**
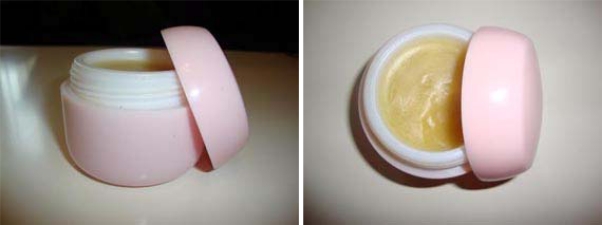
Special Bosnian Balm – “Mehlem”

**Tab. 1.** List of plants used in traditional medicine of the Village of Prokoško Lake

**Botanical taxa & Family**	**Local name**	**Plant part used**	**Preparation & Medicinal use**
*Abies alba* Mill. (Pinaceae)	Jela	resin	**E**: Balm (M) for skin injuries, skin rash, psoriasis and eye injuries.
*Achillea collina s.l.* Becker ex Rchb. (Asteraceae)	Hajdučica, Kunica, Sporiš	leaves	**I**: Tea (M) for bedwetting by children. Decoct (M) for blood purification and as roborantium for strengthening the corpus. **E**: Balm (M) for skin injuries, skin rash and psoriasis.
*Achillea collina × Achillea nobilis* (Asteraceae)	Hajdučica, Kunica, Sporiš	leaves	**I**: Tea (M) for bedwetting by children. Decoct (M) for blood purification and as roborantium for strengthening the corpus. **E**: Balm (M) for skin injuries, skin rash and psoriasis.
*Achillea nobilis* L. (Asteraceae)	Hajdučica, Kunica, Sporiš	leaves	**I**: Tea (M) for bedwetting by children. Decoct (M) for blood purification and as roborantium for strengthening the corpus. **E**: Balm (M) for skin injuries, skin rash and psoriasis.
*Acorus calamus* L. (Acoraceae)	Iđirot	roots	**I**: Tea (SC) for gastro-intestinal spasms and flatulence. Tincture (SC) for vertigo.
*Aesculus hippocastanum* L. (Sapindaceae)	Divlji kesten	buds flowers fruits	**I**: Tincture (SC) for hemorrhoids, varicose veins, blood circulation disorders, intestinal and pulmonary ailments. Tea (SC) for rheumatism. **E**: Fluid unction (M) for rheumatism. Oil (M) for burns. Ointment (M) for varicose veins.
*Agrimonia eupatoria* L. (Rosaceae)	Petrovac	aerial parts	**I**: Tea (M) for spasms.
*Allium ursinum* L. (Alliaceae)	Medvjeđi luk	bulb	**I**: Decoct (SC) for pulmonary diseases.
*Arctium lappa* L. (Asteraceae)	Čičak	roots	**I**: Tea (SC) for digestive ailments, blood purification, internal ulcers, rheumatism and increased diuresis. **E**: Balm (M) for skin injuries.
*Arctium tomentosum* Mill. (Asteraceae)	Čičak	roots	**I**: Tea (SC) for digestive ailments, blood purification, internal ulcers, rheumatism and increased diuresis. **E**: Balm (M) for skin injuries.
*Arnica montana* L. (Asteraceae)	Brđanka	flowers roots whole plant	**I**: Tincture (SC) for strengthening the vocal bands and sickness. Tincture (M) fro earache. Tea (M) for bedwetting by children. Tea (SC) for strengthening the nerves. **E**: Collar (SC) for stomachache and uterine pain.
*Artemisia absinthium* L. (Asteraceae)	Pelin	aerial parts	**I**: Tea (M) for stomachache and cough. Powder (M) for stomachache and stomach spasms.
*Carlina acaulis* L. (Asteraceae)	Vilino sito	roots	**E**: Balm (M) for skin injuries.
*Centaurium erythraea* Rafn (Gentianaceae)	Kičica	aerial parts	**I**: Tea (M) for cough and regulation of menstruation. Tea (SC) for influenza, ague and headache. Decoct (SC) for stomach ailments. Decoct (M) for blood purification. Powder (SC) for digestive ailments.
*Cetraria islandica* Achr. (Cladoniaceae)	Islandski lišaj	lichen	**I**: Decoct (SC) for improving of blood picture and pulmonary diseases. Sirup (SC) for cough and croakiness.
*Euphrasia rostkoviana* Hayne (Orobanchaceae)	Vidac	leaves aerial parts	**E**: Balm (M) for eye injuries. Tea (SC) for eye inflammations. Tea (M) for eye inflammations.
*Fragaria vesca* L. (Rosaceae)	Šumska jagoda	fruits	**I**: Macerate (SC) as roborantium for strengthening the corpus and for renal and gall bladder stones and jaundice. Fresh fruits (SC) for improving of blood picture.
*Frangula alnus* Mill. (Rhamnaceae) Rujevina		bark	**I**: Tea (M) for spasms. Decoct (SC) for strengthening the uterine musculature.
*Geum montanum* L. (Asteraceae)	Planinski čukundjed	roots	**I**: Tea (SC) as roborantium for strengthening the corpus.
*Hypericum montanum* L. (Hypericaceae)	Planinski kantarion	aerial parts flowers	**I**: Decoct (SC) for urinary tract inflammations. Decoct (M) as expectorant, for blood purification and as roborantium for strengthening the corpus. Tea (M) for blood purification. **E**: Oil (M) for burns. Balm (M) for rheumatism.
*Hypericum perforatum* L. (Hypericaceae)	Kantarion, Gospina trava	aerial parts flowers	**I**: Decoct (SC) for urinary tract inflammations. Decoct (M) as expectorant, for blood purification and as roborantium for strengthening the corpus. Tea (M) for blood purification. **E**: Oil (M) for burns. Balm (M) for rheumatism.
*Matricaria discoidea* DC. (Asteraceae)	Kamilica	flowers	**I**: Tea (M) for stomachache, throat inflammations and regulation of menstruation. Decoct (SC) for blood purification. Tea (SC) and powder (M) for stomachache and stomach spasms. Powder (SC) for hemorrhoids. **E**: Collar (SC) for stomachache. Tea (SC) for pain. Oil (M) for burns. Tea (M) for eye inflammations.
*Melissa officinalis* L. (Lamiaceae)	Matičnjak	leaves	**I**: Tea (M) for spasms. M for restlessness and nervousness.
*Mentha arvensis* L. (Lamiaceae)	Poljska nana	leaves	**I**: Tea (M) for spasms and regulation of menstruation.
*Mentha longifolia* (L.) Huds. ssp.	Konjski	leaves	**I**: Tea (M) for spasms and regulation of menstruation.
*longifolia* (Lamiaceae)	bosiljak, Dugolisna nana		
*Mentha pulegium* L. (Lamiaceae)	Veremuša	leaves	**I**: Tea (M) for spasms and regulation of menstruation.
*Olea europea* L. (Oleaceae)	Maslina	fruits	**E**: Oil (M) for burns. Fluid unction (M) for rheumatism. Balm (M) for skin injuries, skin rash, psoriasis, eye injuries and rheumatism. Ointment (M) for varicose veins, bone fractures and burns.
*Picea abies* (L.) H.Karst. (Pinaceae)	Smreka	resin	**E**: Balm (M) for skin injuries, skin rash, psoriasis and rheumatism.
*Picea glauca* (Moench) Voss (Pinaceae)	Bijela smreka	resin	**E**: Balm (M) for skin injuries, skin rash, psoriasis and rheumatism.
*Plantago lanceolata* L. (Plantaginaceae)	Muška bokvica	leaves	**I**: Decoct (M) for blood purification. **E**: Balm (M) for skin injuries, skin rash and psoriasis. Fresh leaves (SC) for maculas.
*Rubus fruticosus agg.* L. (Rosaceae)	Kupina	fruits leaves roots	**I**: Fresh pressed juice (SC) as roborantium for strengthening the corpus. Tea (M) for throat inflammations. Tea (SC) for throat inflammations. Fresh fruits (SC) for digestive ailments. Macerate (M) as roborantium for strengthening the stomach. Sirup (M) for high fever. Decoct (SC) to stop menstruation in menopause. **E**: Powder (SC) for macules.
*Salvia grandiflora* L. (Lamiaceae)	Velika žalfija, Velika kadulja	aerial parts flowers leaves	**I**: Tea (M) for stomachache and throat inflammations. Decoct (SC) for blood purification. Tea (SC) and powder (M) for stomachache and stomach spasms. Tea (SC) for influenza. Tincture (SC) for sedation. Decoct (M) for blood purification and as roborantium for strengthening the corpus.
*Salvia officinalis* L. (Lamiaceae)	Žalfija, Kadulja	aerial parts flowers leaves	**I**: Tea (M) for stomachache and throat inflammations. Decoct (SC) for blood purification. Tea (SC) and powder (M) for stomachache and stomach spasms. Tea (SC) for influenza. Tincture (SC) for sedation. Decoct (M) for blood purification and as roborantium for strengthening the corpus.
*Satureja montana* L. (Lamiaceae)	Bijeli vrijesak	aerial parts	**I**: Tea (SC) for blood diseases and anemia. Tea (M) for blood purification.
*Satureja subspicata* Bartl. ex. Vis. (Lamiaceae)	Crveni vrijesak	aerial parts	**I**: Tea (SC) for blood diseases and anemia. Tea (M) for blood purification.
*Silene vulgaris* (Moench) Garcke (Caryophyllaceae)	Prstopuca	aerial parts	**E**: Tea (SC) for female disorders.
*Stachys officinalis* (L.) Trev. (Lamiaceae)	Čistac	aerial parts	**I**: Tea (SC) for blood purification.
*Symphytum officinale* L. (Boragniceae)	Gavez	leaves roots	**I**: Fresh leaves (SC) for blood purification. Tea (SC) for stomach ulcers. **E**: Fresh pressed juice (SC) for hemostasis. Decoct (SC) for wounds. Ointment (M) for bone fractures and burns.
*Teucrium arduini* L. (Lamiaceae)	Arduinijeva iva	aerial parts	**I**: Tea (SC) for gastro-intestinal ailments.
*Teucrium chamaedrys* L. (Lamiaceae)	Podubica	aerial parts	**I**: Tea (M) for spasms.
*Teucrium montanum* L. (Lamiaceae)	Trava Iva	aerial parts	**I**: Tea (M) for spasms. Tea (M) for blood purification. **E**: Balm (M) for rheumatism.
*Urtica dioica* L. (Urticaceae)	Žara, Velika kopriva	aerial parts buds leaves whole plant	**I**: Sirup (M) for anemia. Decoct (SC) for anemia, blood purification and renal ailments. Decoct (M) for blood purification and asthma. Fresh pressed juice (SC) for anxiety. **E**: Balm (M) and fresh plant (SC) for rheumatism.
*Urtica galeopsifolia* Wierzb. ex Opiz (Urticaceae)	Lažna kopriva	aerial parts buds leaves whole plant	**I**: Sirup (M) for anemia. Decoct (SC) for anemia, blood purification and renal ailments. Decoct (M) for blood purification and asthma. Fresh pressed juice (SC) for anxiety. **E**: Balm (M) and fresh plant (SC) for rheumatism.

**I**…internally; **E**…externally; SC…single compound; M…mixture.
